# Exploring salt tolerance mechanisms using machine learning for transcriptomic insights: case study in *Spartina alterniflora*

**DOI:** 10.1093/hr/uhae082

**Published:** 2024-03-28

**Authors:** Zhangping Huang, Shoukun Chen, Kunhui He, Tingxi Yu, Junjie Fu, Shang Gao, Huihui Li

**Affiliations:** State Key Laboratory of Crop Gene Resources and Breeding, Institute of Crop Sciences, Chinese Academy of Agricultural Sciences (CAAS), Beijing 100081, China; Nanfan Research Institute, CAAS, Sanya, Hainan 572024, China; State Key Laboratory of Crop Gene Resources and Breeding, Institute of Crop Sciences, Chinese Academy of Agricultural Sciences (CAAS), Beijing 100081, China; Nanfan Research Institute, CAAS, Sanya, Hainan 572024, China; Hainan Seed Industry Laboratory, Sanya, Hainan 572024, China; State Key Laboratory of Crop Gene Resources and Breeding, Institute of Crop Sciences, Chinese Academy of Agricultural Sciences (CAAS), Beijing 100081, China; Nanfan Research Institute, CAAS, Sanya, Hainan 572024, China; State Key Laboratory of Crop Gene Resources and Breeding, Institute of Crop Sciences, Chinese Academy of Agricultural Sciences (CAAS), Beijing 100081, China; Nanfan Research Institute, CAAS, Sanya, Hainan 572024, China; State Key Laboratory of Crop Gene Resources and Breeding, Institute of Crop Sciences, Chinese Academy of Agricultural Sciences (CAAS), Beijing 100081, China; State Key Laboratory of Crop Gene Resources and Breeding, Institute of Crop Sciences, Chinese Academy of Agricultural Sciences (CAAS), Beijing 100081, China; Nanfan Research Institute, CAAS, Sanya, Hainan 572024, China; State Key Laboratory of Crop Gene Resources and Breeding, Institute of Crop Sciences, Chinese Academy of Agricultural Sciences (CAAS), Beijing 100081, China; Nanfan Research Institute, CAAS, Sanya, Hainan 572024, China

## Abstract

Salt stress poses a significant threat to global cereal crop production, emphasizing the need for a comprehensive understanding of salt tolerance mechanisms. Accurate functional annotations of differentially expressed genes are crucial for gaining insights into the salt tolerance mechanism. The challenge of predicting gene functions in under-studied species, especially when excluding infrequent GO terms, persists. Therefore, we proposed the use of NetGO 3.0, a machine learning-based annotation method that does not rely on homology information between species, to predict the functions of differentially expressed genes under salt stress. *Spartina alterniflora*, a halophyte with salt glands, exhibits remarkable salt tolerance, making it an excellent candidate for in-depth transcriptomic analysis. However, current research on the *S. alterniflora* transcriptome under salt stress is limited. In this study we used *S. alterniflora* as an example to investigate its transcriptional responses to various salt concentrations, with a focus on understanding its salt tolerance mechanisms. Transcriptomic analysis revealed substantial changes impacting key pathways, such as gene transcription, ion transport, and ROS metabolism. Notably, we identified a member of the *SWEET* gene family in *S. alterniflora*, *SA_12G129900.m1*, showing convergent selection with the rice ortholog *SWEET15*. Additionally, our genome-wide analyses explored alternative splicing responses to salt stress, providing insights into the parallel functions of alternative splicing and transcriptional regulation in enhancing salt tolerance in *S. alterniflora*. Surprisingly, there was minimal overlap between differentially expressed and differentially spliced genes following salt exposure. This innovative approach, combining transcriptomic analysis with machine learning-based annotation, avoids the reliance on homology information and facilitates the discovery of unknown gene functions, and is applicable across all sequenced species.

## Introduction

To discern the potential functions of genes identified through transcriptomic analyses, researchers often turn to functional enrichment analyses employing tools such as the Gene Ontology (GO) database. As the most comprehensive and widely adopted model for describing gene functions, the GO database serves as a cornerstone for functional annotation. When dealing with large quantities of protein sequences, such as those generated from a reference genome assembly for an uncharacterized species, the typical approach involves analyzing protein sequence features to predict functions based on sequence similarity to known proteins. In the pursuit of accurate annotation, researchers often integrate complementary data sources, including protein structure or protein–protein interaction data [1, 2]. However, achieving precise annotations on a whole-genome scale remains a formidable challenge. One contributing feature is the exclusion of less frequent GO terms, constituting ~75% of all annotations in the context of Critical Assessment of Functional Annotation (CAFA), a standard for evaluating the accuracy of protein functional annotation predictions [3].

Protein language models, falling under the umbrella of machine learning (ML), leverage existing protein sequence and structural information to establish a model through ML algorithms. This model is then employed to predict the structure and function of proteins with unknown characteristics. An illustrative instance of this approach is the application of the natural language processing paradigm of pre-training [4] to construct self-supervised protein language models trained on extensive sequence datasets [5, 6]. These algorithms are specifically designed to predict protein structures and functions by utilizing available protein sequences and structural data. In the context of most protein language models, the primary task involves predicting the next amino acid within a sequence, generating protein embeddings that can subsequently be generalized across various tasks [7]. Despite the widespread creation of several protein language models, limited research has been conducted in the field of molecular biology.

Halophytes, constituting ~2% of terrestrial plants, have evolved mechanisms to thrive in saline environments [8], employing structures such as salt glands and salt bladders [9, 10]. Among these halophytes, *Spartina alterniflora*, a member of the Poaceae family, stands out with its distinctive salt gland structure and remarkable salt tolerance. It has developed a potent salt exclusion system enabling its growth in extreme saline conditions [11], and thus in coastal mudflat areas and other saline alkali lands. *Spartina alterniflora*’s salt tolerance, driven by its unique adaptations, positions it as a promising candidate for enhancing salt tolerance and yield in crop species through genetic engineering. Previous studies, exemplified by the ectopic expression of *SaADF2*, *SaSRP3-1*, or *SaVHAc1* in rice (*Oryza sativa* L.), have demonstrated the potential of *S. alterniflora* genes in improving salt tolerance and increasing yield [12, 13]. As salt tolerance becomes increasingly critical for crop genetic improvement in response to dynamic environmental changes, understanding the molecular underpinnings of *S. alterniflora*’s high salt tolerance is imperative.

Despite the potential applications, the molecular mechanisms governing *S. alterniflora*’s outstanding salt tolerance remain largely unexplored. Recent years have witnessed the utilization of transcriptomic profiles to uncover salt-responsive pathways and genes in various plants. Transcriptomic analyses of *Nitraria tangutorum*, rice, *Arabidopsis thaliana*, maize (*Zea mays*), and *Zygophyllum xanthoxylum* have elucidated salt stress-responsive genes and pathways, shedding light on the molecular intricacies of the salt stress response [14–19]. Notably, transcriptome sequencing emerges as a suitable approach to unravel the salt tolerance mechanisms inherent in *S. alterniflora*.

**Figure 1:**
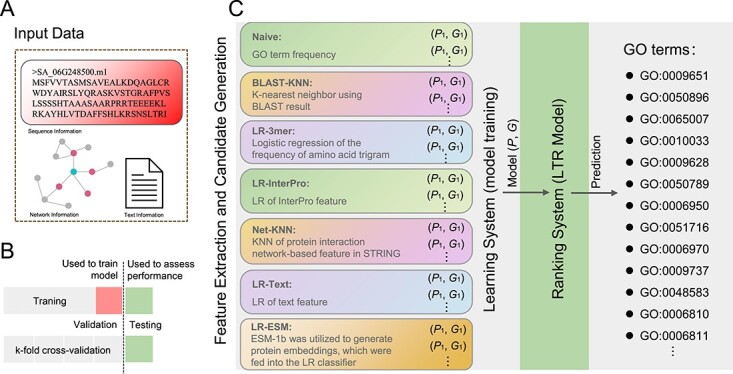
Workflow of the NetGO 3.0 framework. **A** Input data types. **B** Training set, test set, and cross-validation process. **C** Model training, model ranking, and GO term prediction. LR denotes logistic regression, KNN denotes *k*-nearest neighbor, and LTR denotes learning to rank.

Our research group has previously employed the deep learning algorithm DeepGOPlus [20] to automatically extract sequence and protein characteristics, facilitating the exploration of the *S. alterniflora* genome for salt-responsive gene families, including the high-affinity K^+^ transporter (*HKT*) family [21]. Experimental validation of the *SaHKT* genes’ functions confirmed the accuracy of our predictions. Despite these advances, a comprehensive overview of the salt-responsive transcriptional landscape in *S. alterniflora* has not been undertaken. In this study we utilized publicly available *S. alterniflora* RNA-seq data [22] and aligned it with our newly assembled *S. alterniflora* genome. Analyzing the *S. alterniflora* transcriptome’s response to multiple NaCl concentrations provided a thorough understanding of dynamic responses to salt stress in this unique species. Leveraging an ML-based protein annotation approach facilitated genome-wide GO analysis of salt-responsive genes, revealing previously unidentified pathways in the salt stress response. Our findings suggest that utilizing ML models for GO annotation of newly assembled genomes could enhance our understanding of functionality compared with relying solely on background files from other species for annotation. Additionally, a comprehensive analysis of differential splicing in response to salt stress was conducted to unveil the involvement of post-transcriptional modifications and key splicing-associated genes that contribute to the unique stress responses of this halophyte. These analyses establish a robust foundation for comprehending the molecular mechanisms associated with stress responses in a highly salt-tolerant species, uncovering candidate genes for future transgenic improvements in key crop plants.

## Results

### Overview of the fundamental principles of NetGO 3.0

Figure 1 illustrates the overall framework of NetGO 3.0. The fundamental concept of this model is to integrate seven component methods within a learning-to-rank (LTR) framework to enhance the performance of automatic function prediction (AFP). Prior to testing proteins, NetGO 3.0 must be trained using a vast collection of instances, which consist of protein sequences, their network information, and associated ground-truth GO annotations (Fig. 1A). Additionally, cross-validation is employed to partition the training and testing sets (Fig. 1B). The seven components of the model utilize their learned parameters to extract features from the protein, resulting in a score feature vector of length 7. For detailed information on the seven-component model, please refer to previous studies [7, 23, 24]. Subsequently, candidate GO terms are input into the LTR model, whose objective is to generate an optimal ranking of GO annotations for all protein pairs in the training data. The final output of NetGO for a query protein is a ranked list of GO terms (Fig. 1C). This approach is applicable to all sequenced species, enabling a deep understanding of their biological functions. In this study we aim to utilize NetGO 3.0 to explore the molecular mechanisms underlying salt tolerance in *S. alterniflora*, thereby demonstrating the power of this ML approach.

### Salt stress triggers changes in salt glands and ion content of *S. alterniflora*

**Figure 2:**
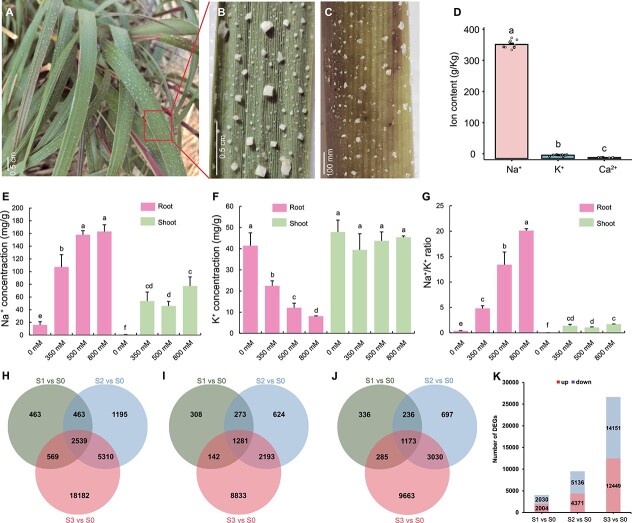
Features of salt glands and identification of DEGs in *S. alterniflora* under salt stress. **A** Coastal salt marsh environment with *S. alterniflora*. Scale bar = 0.5 cm. **B**, **C** Representative images of leaf (**B**) and stem (**C**) of *S. alterniflora* in coastal salt marshes. Scale bars in **B** and **C** are 0.5 cm and 100 mm, respectively. **D** Leaf surface exudate composition from plants grown in a coastal salt marsh. **E**–**G** Root and shoot Na^+^ (**E**) and K^+^ (**F**) contents and Na^+^/K^+^ ratios (**G**) in *S. alterniflora* seedlings (*n* = 5). Values are mean ± standard deviation. Different letters indicate significant differences at *P* < 0.05 (Duncan’s multiple range test). **H**–**J** Unique and overlapping (**H**) DEGs, (**I**) upregulated DEGs, and (**J**) downregulated DEGs among three NaCl treatments compared with the 0 mM NaCl control. (**J**) Summary of up- and downregulated DEGs after treatment with each salt concentration.

Being a recretohalophyte, *S. alterniflora* exhibits typical salt glands that actively secrete excess salt through secretory pores, leading to the visible accumulation of salt crystals on the stem and leaf surfaces of plants in coastal marshes of Dongying, Shandong Province, China (Fig. 2A–C). To quantify ion concentrations in leaf tissue, we conducted inductively coupled plasma mass spectrometry (ICP-MS) analysis, revealing a notably higher Na^+^ concentration compared with other ions, with an average of 366.03 ± 12.87 g/kg (Fig. 2D). In contrast, K^+^ and Ca^2+^ exhibited average concentrations of 10.55 ± 2.16 and 2.93 ± 0.49 g/kg, respectively.

To investigate ion transport activity in *S. alterniflora*, we subjected seedlings to increasing salinity, ranging from 0 to 800 mM NaCl in a hydroponic medium over a 24-h treatment period. Our analysis revealed a gradual increase in Na^+^ contents from 0 to 800 mM in root samples, with Na^+^ concentration consistently higher in roots than in shoots across all NaCl treatments (Fig. 2E). In contrast, K^+^ contents exhibited a significant decrease in roots but showed no apparent change in shoots (Fig. 2F). Consequently, Na^+^/K^+^ ratios progressively and significantly increased along with escalating NaCl concentrations in both roots and shoots (Fig. 2G). These findings underscore the impact of salt stress on ion transport dynamics in *S. alterniflora.*

**Figure 3:**
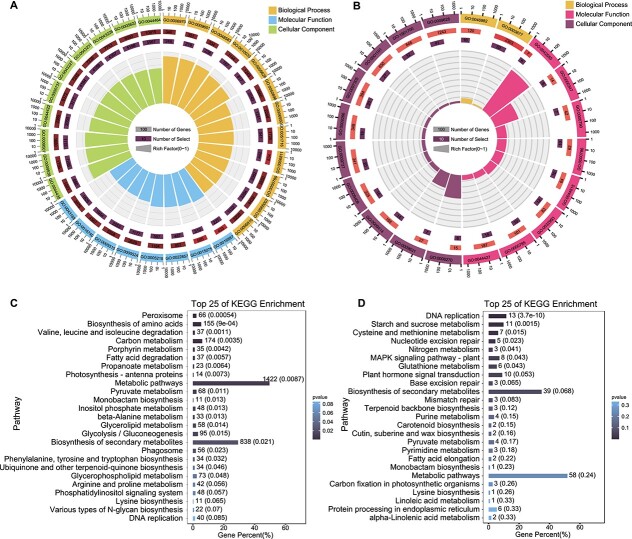
Functional enrichment analyses of DEGs in response to salt stress. **A**, **B** GO enrichment analysis of DEGs in (**A**) *S. alterniflora* and (**B**) rice using NetGO 3.0. **C**, **D** KEGG enrichment analysis of DEGs in (**C**) *S. alterniflora* and (**D**) rice under salt stress, displaying the 25 most enriched biochemical pathways.

### Salt stress significantly alters *S. alterniflora*’s transcriptome

To unravel transcriptomic responses to environmental stress, we conducted an analysis using transcriptomic data from a prior study involving *S. alterniflora* seedlings subjected to 0, 350, 500, or 800 mM NaCl for 24 h, denoted as S0, S1, S2, and S3, respectively [22]. The investigation revealed a total of 28 721 differentially expressed genes (DEGs) across the three treatment groups, including a core set of 2539 genes exhibiting differential expression in all three groups (Fig. 2H). Among these, 1281 were upregulated and 1173 were downregulated at all three concentrations, while the remaining genes displayed inconsistent directionality (Fig. 2I–J). Notably, the total number of DEGs increased proportionally with the salt concentration, resulting in 4034, 9507, and 26 600 DEGs in S1, S2, and S3, respectively. This comprised 2004, 4371, and 12 449 upregulated DEGs and 2030, 5136, and 14 151 downregulated DEGs in the respective groups (Fig. 2K). These findings underscored the significant transcriptional changes induced by salt stress.

To explore potential similarities in gene expression patterns between *S. alterniflora* and other plants under salt stress, we conducted a comparative analysis of transcriptome data from rice exposed to salt treatment in the same study [22]. Our comparison revealed a total of 582 DEGs identified at 1, 5, and 24 h after 300 mM NaCl stress in rice (Supplementary Data Table S1). To explore whether *S. alterniflora* and rice exhibit similar expression profiles in response to salt stress, we aligned 28 721 *S. alterniflora* DEGs with rice DEGs, identifying 341 orthologs (Supplementary Data Table S2). Of the 341 orthologous genes, 22 encode transcription factors (TFs), including ERF (ethylene-responsive factor), NAC (NAM, ATAF1/2, CUC1/2), and WRKY, among others, while 16 are annotated as functional proteins, including those encoding SWEET (sugars will eventually be exported transporter), PEBP (phosphatidyl ethanolamine-binding protein), BBX (B-box zinc finger protein), and other proteins.

### Machine learning uncovers critical biological functions associated with salt tolerance in *S. alterniflora*

To gain deeper insights into the specific functions of *S. alterniflora* DEGs, we employed NetGO 3.0, an ML-based protein language model, to identify commonalities in the annotations of 28 721 salt-responsive DEGs in *S. alterniflora* and 582 salt-responsive DEGs in rice in response to salt stress.

GO analysis highlighted significant enrichment of *S. alterniflora* DEGs in various biological process terms, including ‘response to stimulus’ (GO:0050896, GO:0009628, and GO:0051716), ‘salt stress’ (GO:0009651), ‘ion transport’ (GO:0006811, GO:0030001, GO:0006812, GO:0043269, and GO:0006813), ‘response to abscisic acid’ (GO:0009737, GO:0071215, GO:0009738, and GO:0009787), and ‘reactive oxygen species metabolism’ (GO:0072593, GO:2000377, and GO:1903409) (Fig. 3A and Supplementary Data Table S3). Similarly, rice DEGs were significantly enriched in ‘response to stimulus’ (GO:0050896, GO:0009628), ‘salt stress’ (GO:0009651), ‘response to abscisic acid’ (GO:0009737), and ‘reactive oxygen species metabolism’ (GO:2000377 and GO:1901700) (Fig. 3B and Supplementary Data Table S4), indicating shared salt response pathways between the two species.

**Figure 4:**
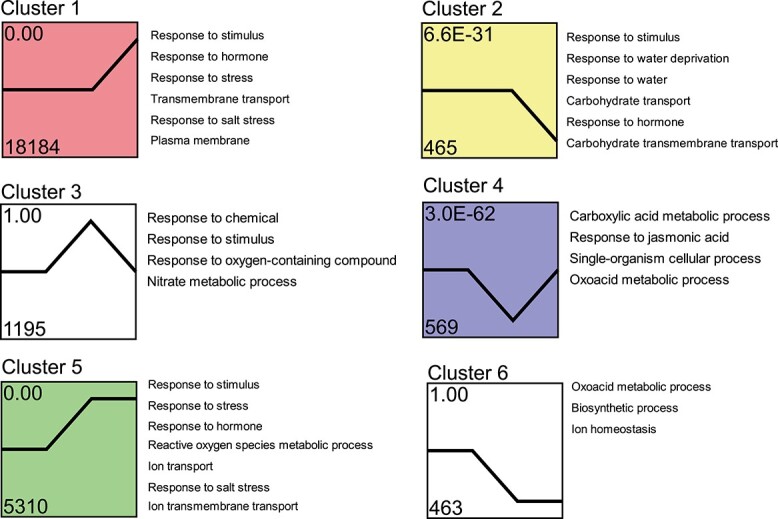
Trend analysis of DEGs. The 28 721 DEGs in *S. alterniflora* responding to salt treatment were clustered into nine profiles, the displayed profiles being the top six in terms of total number. Each row in the figure represents the clustering results for each NaCl concentration. Legends on the right indicate the GO enrichment results for the corresponding profiles. The number above the upper left of each box denotes different trend clusters. The number at the bottom of the box indicates the gene numbers enriched in this profile, and the number at the top of the box represents the *P*-value significance of the cluster. Different colors in clusters 1, 2, 4, and 5 denote significant expression patterns (*P* < 0.05).

Further exploration using the Kyoto Encyclopedia of Genes and Genomes (KEGG) database unveiled that *S. alterniflora* DEGs were associated with 136 KEGG biochemical pathways (Supplementary Data Table S5), primarily in metabolism, especially specialized metabolite biosynthesis (e.g. ko01100). While the relative enrichment factor differed between rice and *S. alterniflora*, rice DEGs were also predominantly enriched in metabolic pathways (Fig. 3C and D)*.* These findings underscored the functional annotation similarities between *S. alterniflora* and rice under salt stress.

To validate the accuracy of the ML model, we performed additional GO annotations using the homologous alignment method. Aligning 28 721 DEGs from *S. alterniflora* to the rice genome and conducting GO analysis for the best matches in the rice genome revealed 12 986 rice homologs. These homologs were mainly enriched in signal transduction pathways but did not show enrichment in the biological processes of ‘ion transport’ or ‘salt stress’ (Supplementary Data Table S6). This comparison highlights the necessity of employing methods, such as NetGO 3.0, that do not rely on homology to known genes for establishing gene functions.

### Trend analysis unveils dynamic changes in salt stress-responsive genes

To comprehend the general expression patterns of DEGs in response to salt stress in *S. alterniflora*, we employed the Short Time-series Expression Miner software (STEM) program to group genes with similar expression profiles across treatment groups. The number of genes exhibited substantial variation among clusters ranged from 8 to 18 184 per cluster (Supplementary Data Fig. S1), excluding the cluster containing only 1 gene. The expression patterns from different clusters demonstrated diverse variations (Fig. 4 and Supplementary Data Fig. S1).

For the purpose of analyzing the common mechanisms underlying the expression patterns, we categorized the expression patterns into three sets: late-up/down response (clusters 1 and 2), transient up/down response (clusters 3, 4, 5, and 6), and gradual up/down response (clusters 7 and 8). The late-up pattern (cluster 1), characterized by genes upregulated in all salt treatment groups, and peaking in S3, was the largest cluster, comprising 18 184 genes (Fig. 4). In contrast, 465 genes exhibited the late-down pattern (cluster 2). Cluster 1 contained 63.31% of the DEGs in *S. alterniflora*, indicating its dominance in gene expression in response to increasing salt concentrations. The transient up/down clusters, featuring genes strongly induced in S2, also encompassed a substantial number of genes (1195 and 569 in the transient up [cluster 3] and transient down [cluster 4] groups, respectively). The other two transient up/down clusters (clusters 5 and 6) comprised genes that were up- or downregulated in S2, maintaining this regulation in S3. The gradual up cluster (cluster 7) consisted of 14 genes, increasing in expression across NaCl concentrations, peaking in S3. Similarly, cluster 8 included eight genes consistently decreasing across salt concentrations, reaching minima in S3 (Supplementary Data Fig. S1). These findings indicated that higher salt concentrations exerted more pronounced effects on these genes*.* Overall, the clustering analysis highlighted late upregulation and transient upregulation as the dominant expression patterns.

To elucidate the primary biological processes and cellular components affected by salt, we conducted a functional annotation analysis of each cluster using NetGO 3.0. This analysis unveiled a diverse array of salt-affected GO categories (Fig. 4). Specifically, clusters 1 and 5 (containing the majority of DEGs, which were upregulated in S3) exhibited over-representation of stress signaling terms, including pathways associated with hormones, reactive oxygen species (ROS), and ion transport. These results correspond to the GO analysis of the DEGs presented in Fig. 3A.

### Salt stress triggers differential expression of genes in *S. alterniflora*

To delve deeper into the expression and functional characteristics of DEGs induced by salt in *S. alterniflora*, our focus shifted to DEGs annotated as components of the salt stress response, ROS metabolism, or ion transport in subsequent analyses*.*

#### Differentially expressed genes associated with the salt stress response

**Figure 5:**
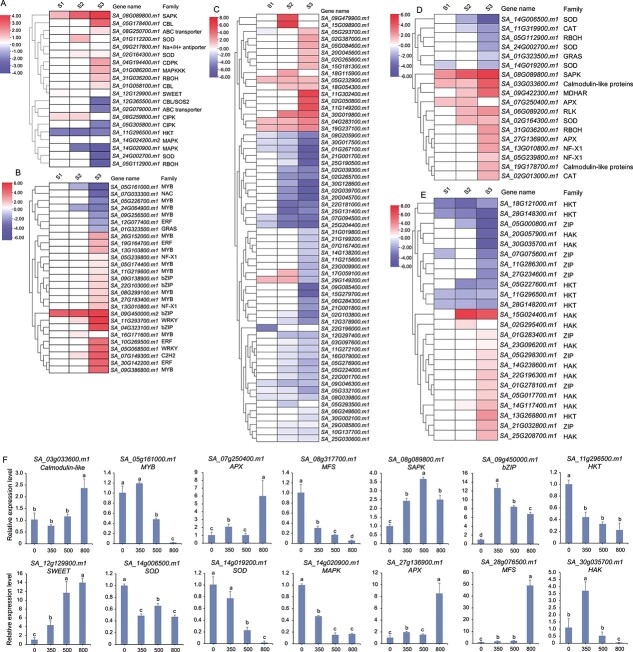
Expression patterns of DEGs with functional annotations ‘salt response’, ‘ROS metabolism’, and ‘ion transport’. **A–E** Expression patterns of DEGs with the GO terms (**A**, **B**) ‘salt response’, (**C**, **D**) ‘ROS [reactive oxygen species] metabolism’, and (**E**) ‘ion transport’ in response to treatment with various salt concentrations. The darker shades of blue indicate lower expression levels, while redder colors represent higher expression levels. (**F**) qRT–PCR analysis of 14 DEGs in response to salt stress. The horizontal axis depicts the varying salt concentration treatments, specifically 0, 350, 500, and 800 mM, while the vertical axis represents the relative expression levels of these genes. The presence of different lower-case letters among the treatments indicates statistically significant differences at a significance level of *P* < 0.05.

A total of 364 DEGs associated with the GO term ‘salt response’ were identified in *S. alterniflora* (GO:0009651; Supplementary Data Table S7). Among them were two genes involved in Na^+^ transport: a sodium transporter gene (*SA_11G296500.m1*), with expression levels negatively correlated with salt concentration, and an Na^+^/H^+^ antiporter gene (*SA_09G217800.m1*), upregulated by high salt concentrations (Fig. 5A). The expression patterns of these genes closely resembled those of their homologs in rice following salt exposure [25, 26]. Additionally, several Ca^2+^-dependent signal transducer genes were identified, including genes encoding a calcium-dependent protein kinase (CDPK), three calcineurin B-like (CBL) proteins, and two calcineurin B-interacting protein kinases (CIPKs), all of which showed significant differential expression after exposure to high salt concentrations. Treatment with 800 mM NaCl upregulated *SA_05G178400.m1* (*CBL*), *SA_01G058100.m1* (*CBL*), and *SA_04G194400.m1* (*CDPK*), and downregulated *SA_12G365500.m1* (*CBL*/*SOS2*) and *SA_05G305800.m1* (*CIPK*). Expression of *SA_08G089800.m1* (stress-activated protein kinase, *SAPK*) was positively correlated with salt concentration, while the expression levels of *SA_11G296500.m1* (*HKT*) and *SA_14G020900.m1* (*MAPK*) were negatively correlated with salt concentration. A total of 27 TFs were identified as involved in the salt stress response, the majority not showing differential expression between S1 and S2. Among these, 19 were only upregulated in S3. The bZIP *SA_09G450000.m2* and the MYB *SA_05G161000.m1* exhibited positive and negative correlations, respectively, with salt concentration (Fig. 5B).

#### Differentially expressed genes associated with reactive oxygen species metabolism

A total of 142 DEGs associated with ROS metabolism were identified (Supplementary Data Table S8), with the majority being secretory peroxidases (66 genes; Fig. 5C and D). Predominantly, these were class III peroxidases (PRXs), known to contribute to salt resistance by regulating peroxidase (POD) activity and ROS contents in peroxisomes [27]. Most secretory peroxidases were downregulated by salt stress, and only 12 were differentially expressed across all three salt treatments. Additionally, six genes associated with ROS scavenging were identified: four superoxide dismutase (*SOD*) genes and two ascorbate peroxidase (*APX*) genes. Among these, three *SOD*s (*SA_14G006500*.*m1*, *SA_24G002700.m1*, and *SA_14G019200.m1*) were downregulated by salt stress, while both *APX*s (*SA_07G250400.m1* and *SA_27G136900.m1*) were upregulated. Expression levels of genes encoding one stress-activated protein kinase (SAPK) (*SA_08G089800.m1*) and one calmodulin-like protein (*SA_03G033600.m1*) were positively correlated with salt stress.

#### Differentially expressed genes associated with ion transport

In our analysis we identified 24 ion transporter genes among the DEGs (Fig. 5E), including 6 *HKT*s, 10 high-affinity K^+^ transporters (*HAK*s)*,* and 8 zinc transporter genes (*ZIP*s). Among the *HAK*s, *SA_20G057900.m1* and *SA_30G035700.m1* were downregulated in S3, while all other *HAK* genes were upregulated in every salt treatment. Only one *HKT* (*SA_13G268800.m1*) was upregulated in S3, as the other *HKT*s were downregulated in this treatment group. We also identified salt-responsive voltage-gated potassium channel genes, including cyclic nucleotide-gated channel (*CNGC*), high-affinity K^+^/K^+^ uptake permease/K^+^ transporter (*HAK/KUP/KT*), gated outwardly-rectifying K^+^ channel (*GORK)*, and stelar K^+^ outward-rectifying channel (*SKOR*) (Supplementary Data Table S9). Some *ZIP*s and sugar transporter genes (*SLC*s) involved in ion transport were also identified as salt-responsive and underwent significant induction by high salt. Nine mitochondrial carrier protein genes, crucial for ion transporter function, were differentially expressed in response to salt treatment; eight of these were upregulated, except for *SA_01G284900.m1*. We found 45 members of the major facilitator superfamily (*MFS*s) in the *S. alterniflora* genome, all of which were induced by salt stress. Expression levels of the *MFS* genes *SA_01G111900.m1*, *SA_28G076500.m1*, and *SA_11G055300.m1* were positively correlated with salt concentration, whereas *SA_08G317700.m1* and *SA_10G103900.m1* were negatively correlated. These findings suggest that ion transport-based responses to salt stress involve more than just Na^+^ and K^+^ transport, and that various types of protein transporters may function synergistically.

To validate the key DEGs, we utilized quantitative real-time PCR (qRT–PCR) to confirm the expression of 14 randomly selected DEGs (Fig. 5F). The results demonstrated that their expression trends under salt stress were consistent with the transcriptome data.

### An *S. alterniflora SWEET* gene contributed to salt tolerance

**Figure 6:**
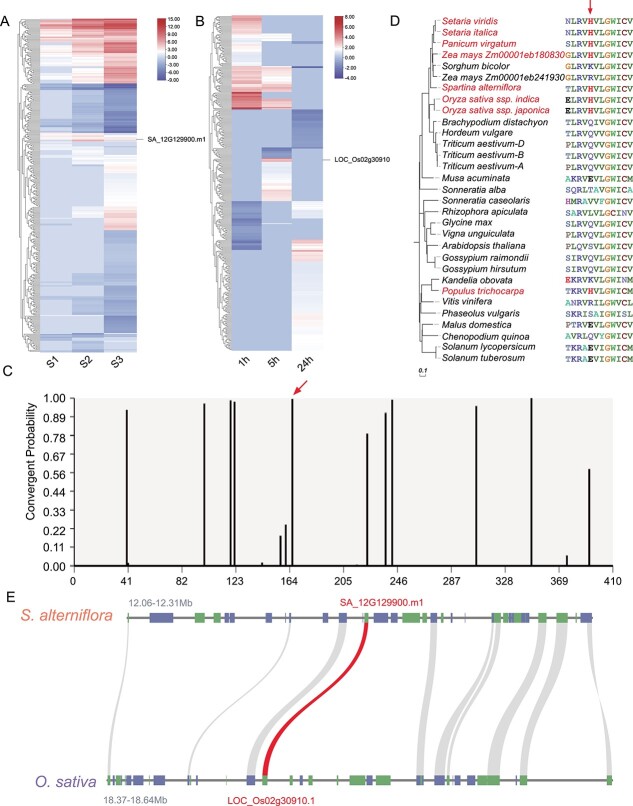
Convergent selection analysis of a SWEET protein in *S. alterniflora* and rice. **A**, **B** Expression patterns of orthologs in (**A**) *S. alterniflora* and (**B**) *O. sativa*. **C** Probability of convergent changes in the SWEET protein sequence between *S. alterniflora* and rice. The red arrow denotes the position in the protein sequence alignment where the divergence occurred (highlighted in red text). **D** Convergent amino acid change (from K/R/Q/E/V/L/T/S to H) in SWEET shared by different species (highlighted in red) at the position indicated by a red arrow. **E** Comparative genomic analysis of syntenic and conserved sequences in the 0.25/0.27-Mb region surrounding *SA_12G129900.m1*/*LOC_Os02g30910.1* (red) in *S. alterniflora* and *O. sativa* ssp. *japonica* cv. ‘Nipponbare.’

As previously mentioned, a total of 341 orthologs of *S. alterniflora* were identified among the rice DEGs (Supplementary Data Table S2). Despite the overall dissimilarity in expression patterns of most orthologous gene pairs (Fig. 6A and B), 41 *S. alterniflora* salt-responsive DEGs displayed similar expression patterns to rice salt-responsive DEGs. These included 33 gene pairs with upregulated expression and 8 gene pairs with downregulated expression (Supplementary Data Table S2). To gain insights into whether there are commonalities among these orthologous gene pairs, we employed Grand Convergence to detect amino acid convergent selection between these orthologous gene pairs. Interestingly, we found convergent selection in an upregulated DEG in *S. alterniflora* (*SA_12G129900.m1*) and its upregulated homolog in rice (*LOC_Os02g30910*/*OsSWEET15*). Specifically, the *S. alterniflora* gene *SA_12G129900.m1*, a member of the SWEET family, exhibited convergent selection at amino acid position 166 (Fig. 6C). Multiple sequence alignment revealed that this residue, histidine (H166), was conserved in homologs across various species, including *O. sativa* ssp. *indica*, *O. sativa* ssp. *japonica*, *Z. mays* (Zm00001eb180830), *Setaria viridis*, *Setaria italica*, *Panicum virgatum*, and *Populus trichocarpa*. However, other amino acids, including arginine (R), lysine (K), and serine (S), were conserved among SWEET proteins in other species (Fig. 6D).

*SA_12G129900.m1* mapped to a region of *S. alterniflora* chromosome 12 (12.06–12.31 Mb) that had undergone a selective sweep and showed synteny with the short arm of chromosome 2 in rice (Fig. 6E). Previous research demonstrated that salt stress significantly induces *OsSWEET15* in rice, modulating sucrose transport and distribution [28]. Considering functional annotations, gene conservation, and expression levels of *SA_12G129900.m1* and its orthologs, we propose that this gene is crucial for salt tolerance in *S. alterniflora*.

### Salt stress triggers alterations in post-transcriptional levels in *S. alterniflora*

**Figure 7:**
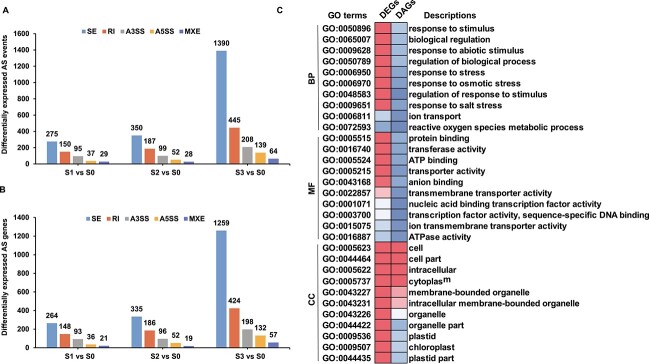
Comparative analysis of DEGs and DAGs in response to salt stress. **A** Differential AS events in salt-treated compared with control plants. **B** Differentially expressed AS genes in salt-treated compared with control plants. **C** Functional enrichment examination of DEGs and DAGs. The heat map displays the enrichment factors of significantly enriched (*P* < 0.05) GO terms.

To comprehensively investigate the alternative splicing (AS) landscape in *S. alterniflora* under high salt conditions, we screened 3548 salt-responsive AS events, corresponding to 2463 genes (Supplementary Data Table S10). Genes undergoing salt stress-responsive AS events were defined as differentially alternative-spliced genes (DAGs), with 586, 716, and 2246 DAGs identified in response to S1, S2, and S3 treatments, respectively. The distribution of AS types was comparable between treatment groups, with exon skip (ES) being the most abundant AS event type, followed by retained intron (RI), alternative 3′ splice site (A3SS), alternative 5′ splice site (A5SS), and mutually exclusive exon (MXE) (Fig. 7A and B). Noteworthy DAGs included important *S. alterniflora* salt-responsive genes (Supplementary Data Table S10), such as TFs, ion transporters, and protein kinases.

Considering that Ser/Arg-rich (SR) proteins are crucial AS regulators in plants [29], we investigated the expression levels and AS of *S. alterniflora SR* genes in response to salt treatment. Only seven *SR* genes were differentially spliced under salt treatment (Supplementary Data Table S11), constituting 14.89% of all *SR* genes in the *S. alterniflora* genome. This was fewer than the differentially spliced *SR* genes observed in wheat and rice in response to salt stress [30, 31], suggesting a limited effect of salt stress on *S. alterniflora SR* genes. To explore the relationship between AS and transcriptional regulation, we compared DEGs and DAGs in response to salt stress. Among the DAGs, there were only 24, 41, and 95 DEGs at S1, S2, and S3, respectively, representing 0.59, 0.43, and 0.36% of all DEGs under these conditions (Supplementary Data Fig. S2). Although AS is known to play important regulatory roles in plants at the post-transcriptional level in response to hostile environmental conditions, *S. alterniflora* underwent relatively few post-transcriptional modifications following salt stress compared with other characterized species.

To understand the biological functions of genes regulated by AS and/or transcriptional modulation in response to salt stress, GO enrichment analyses were performed. While transcriptional activity appeared to be the primary driver of salt stress responses, with very few GO terms enriched among the DAGs, some common terms related to plant development and stress responses were enriched in both gene sets (Fig. 7C). Notably, key terms enriched in the DEGs, such as ‘response to stimulus’, ‘response to stress’, ‘response to salt stress’, ‘ion transport’, and ‘reactive oxygen species metabolic process’ were not enriched in the DAGs. Some GO terms common to both plant development and stress responses were enriched in both gene sets (including the cellular compartment terms ‘cell’, ‘cell part’, and ‘cytoplasm’). These findings suggest that some salt-sensitive pathways in *S. alterniflora* are subject to dual regulation at both the transcriptional and post-transcriptional levels.

## Discussion

### Machine learning is a powerful tool for annotating genes with Gene Ontology terms

Conventional GO annotation faces limitations due to its reliance on existing annotation information, which is often derived from experimental data available for only a subset of genes and species. This complicates large-scale analyses and comparisons across diverse species. ML offers a solution to these challenges by integrating and comparing results from different experimental datasets and annotations, enhancing the accuracy and repeatability of annotation-based outcomes. In our study, we employed the ML-based annotation model NetGO 3.0 to annotate genes from the relatively under-studied species *S. alterniflora*. By utilizing this ML approach, we are able to uncover previously unrecognized biological narratives, and the method is applicable to all sequenced species, making it highly usable for exploring gene functions.

The enriched GO annotations for salt-responsive genes, identified through ML, predominantly encompassed biological processes related to osmotic stress, signal transduction, and ion transport. A comparison between the ML-based method and an ortholog-based annotation method revealed substantial differences in enriched functions among salt-responsive DEGs. This discrepancy suggests that GO annotations based on gene homology may not accurately capture gene functions in the context of salt stress response. Overall, the use of ML models for GO annotation provides several advantages over traditional methods. These include increased accuracy, the potential for automation, and the ability to conduct large-scale annotations, such as whole-genome annotations. These factors significantly enhance gene annotation efficiency, enabling accurate annotations of entire genomes, particularly for species that have not been extensively characterized. In our study, the application of an ML-based annotation model allowed us to effectively explore the salt-responsive transcriptome of *S. alterniflora*, shedding light on the biological processes associated with salt stress resistance in this uniquely salt-tolerant species.

### Salt stress triggers intricate transcriptional changes in *S. alterniflora*

Transcriptional regulation plays a crucial role in enabling higher plants to survive salt stress. However, understanding the key genes and regulatory pathways that control plant growth and development at both the transcriptional and post-transcriptional levels in salt-stressed halophytes remains limited. In this study, our objective was to identify key salt-responsive genes in *S. alterniflora* and to elucidate the molecular mechanisms involved in the salt stress response. We employed transcriptomic and ML-based gene annotation methods to achieve a comprehensive understanding of the salt stress response in this halophytic species.

Plants exhibit adaptive responses to various environmental stresses, including salt stress, through the regulation of transcript levels in specific genes associated with osmotic stress, signal transduction, transcriptional regulation, and ion transport [32–35]. In our study, we aimed to identify the processes involved in regulating salt stress responses, by analyzing the GO terms of DEGs in *S. alterniflora* under varying levels of salt stress. Notably, we observed distinct differences in the enriched GO terms among salt-responsive DEGs in *S. alterniflora* compared with well-characterized model plant species.

For instance, in *A. thaliana*, salt-responsive DEGs are primarily associated with phytohormone pathways, transcriptional regulation, general metabolism, energy production, and cell wall modification [36]. In maize hybrids, salt-responsive DEGs are mainly linked to processes related to photosynthesis and redox reactions [16]. However, in *S. alterniflora*, salt-responsive DEGs were notably enriched in biological process terms such as ‘response to stimulus’, ‘response to salt stress’, ‘ion transport’, and ‘ROS metabolism’. This pattern aligns with the salt-responsive DEGs observed in the alkaline-tolerant grass species *Puccinellia nuttalliana*, but diverges from the responses of rice, maize, and *A. thaliana* to salt exposure [17, 37–39].

The distinct response in *S. alterniflora* may be attributed to its natural habitat in saline environments, indicating evolutionary adaptations that provide a greater basal capacity to cope with external salt stress. This inherent capacity is further bolstered by ion transporter genes, enhancing the plant’s ability to withstand salt stress. Remarkably, we observed more complex changes in *S. alterniflora* gene expression with higher salt concentrations compared with lower concentrations. This complexity is reflected in the increased number of DEGs, greater transcript diversity, and activation of a larger number of pathways. However, the Na^+^ content of seedlings treated with 800 mM NaCl did not differ significantly from those treated with 500 mM NaCl. This resilience is attributed to *S. alterniflora* adaptive mechanisms enabling survival in high-salt environments. These mechanisms regulate ion transporter activities, control ion absorption and excretion, and modulate intracellular osmolality to balance external salt stress. Therefore, halophytes maintain intracellular ion homeostasis despite increasing external salt concentrations. Additionally, gene expression changes do not always directly correlate with altered ion content; some genes may respond to environmental fluctuations without directly regulating ion homeostasis [11]. The findings suggest that the identification of key salt tolerance genes in halophytes, such as *S. alterniflora* and *P. nuttalliana*, may necessitate the application of more extreme experimental conditions.

The expression of numerous calcium-dependent signaling genes is modulated in response to salt stress. For instance, upon exposure to high salt concentrations, *CDPK*, *CBL*, and *CIPK* genes exhibited significant differential expression patterns, with some genes being upregulated and others downregulated (Fig. 4A). High doses of Na^+^ trigger changes in the expression of Ca^2+^-dependent genes in cells due to shared transport and signaling pathways with Ca^2+^ [40]. This occurs when Na^+^ enters cells, competing with Ca^2+^ for binding sites, disrupting calcium homeostasis. This modulation, such as changes in CBLs, affects Ca^2+^ levels and enhances plant salt tolerance [41–43]. The differential expression of these genes is complex, involving multiple factors and mechanisms, likely as a cellular response to maintain homeostasis under salt stress. For instance, CNGCs, voltage-gated ion channels, form ABA-activated Ca^2+^ channels. *CNGC10* negatively regulates salt tolerance in *Arabidopsis*, while *AtCNGC19* and *AtCNGC20* are upregulated in salinity stress [44, 45]. Microarray profiling in rice revealed differential expression of cation/Ca^2+^ exchangers like *OsCCX1*, *OsCCX2*, and *OsCCX3* under Ca^2+^ deficiency [46]. Global expression analysis showed differential expression of Ca^2+^ transporter genes in salinity-stressed rice [47]. In summary, Ca^2+^-dependent gene expression varies under salt conditions.

In our comparative analysis of the transcriptomes of the halophyte *S. alterniflora* and the non-halophyte rice after exposure to salt stress, a homologous gene pair was identified with both members showing upregulation: *SA_12G129900.m1* and *OsSWEET15*. Both genes belong to the SWEET family of transporters, known for their involvement in various plant growth and developmental processes, including senescence, seed and pollen development, and grain filling in crop species such as rice and maize [48, 49]. However, the functions of SWEET transporters in abiotic stress responses, particularly salt stress, have not been thoroughly characterized. Our analysis revealed that *SA_12G129900.m1* and its rice ortholog *OsSWEET15* underwent convergent selection during evolution. This finding is consistent with evidence suggesting convergent selection in other *SWEET* genes, such as *ZmSWEET1b* in maize and its rice ortholog *OsSWEET1b*. *ZmSWEET1b* has been shown to play a crucial role in maize plant development and salt stress responses, potentially influencing the transcriptional abundance of genes responsible for Na^+^ efflux from roots to the rhizosphere [50]. Understanding the evolution of *SWEET* genes among domesticated crop species provides valuable insights into potential targets for molecular design breeding. This approach aims to develop plants with optimized sugar transport activity and enhanced salt tolerance.

### Post-transcriptional modifications may not effectively enhance salt tolerance in *S. alterniflora*

AS has emerged as a potential target for genetic manipulation to regulate plant environmental fitness, given its role as a key regulator of plant function. Developmental genes with stress-induced spliceoforms have been identified as candidate AS genes for this purpose, contributing to environmental stress tolerance [29]. In the case of *S. alterniflora*, we conducted a comprehensive analysis of its transcriptome in response to salt stress to identify alternative forms of salt-responsive gene transcripts and assess their potential for genetic manipulation. Our analysis revealed a total of 3548 salt-induced AS events, corresponding to 2463 genes in *S. alterniflora.* Among these, seven *SR* genes, known to regulate splicing events, exhibited distinct AS events in response to salt stress. However, it is noteworthy that none of these *SR* genes were differentially expressed after exposure to high concentrations of salt. This observation aligns with similar findings in salt-stressed cotton, suggesting a preferential regulation of splicing regulators in plants at the post-transcriptional level [51].

In addition to the known AS events, there is a likelihood of numerous unidentified or unvalidated AS events that may play a role in stress responses. For instance, a genome-wide AS analysis in rice identified 764 significant genotype-specific splicing events under salt stress conditions [38]. In wheat, 1141 genes exhibited significant changes in AS after exposure to salt stress [52]. Furthermore, in *G. davidsonii* 1287 and 1228 differential AS events were identified in the roots and leaves, respectively [53]. However, the number of AS genes in *S. alterniflora* was comparatively lower than in other species under salt stress conditions. This discrepancy could be attributed to the adaptation of *S. alterniflora* to extreme saline environments, resulting in many salt-tolerance genes being basally active rather than stress-responsive. Previous studies have suggested that AS and transcriptional regulation are parallel processes that operate independently in response to environmental stressors. Little overlap has been identified between DEGs and DAGs in response to biotic and abiotic stressors in *A. thaliana*, wheat, and *Nicotiana attenuata* [52, 54–56]. Similarly, in *S. alterniflora*, we found that only 0.36–0.59% of DEGs in response to salt stress also underwent AS. GO enrichment analyses revealed that several abiotic stress-responsive GO terms were significantly enriched in DEGs, including ‘response to stimulus’, ‘response to abiotic stimulus’, ‘response to stress’, ‘response to osmotic stress’, and ‘response to salt stress’. However, very few of these GO terms overlapped with those enriched in DAGs. Consequently, targeting AS to enhance salt tolerance may not be a promising strategy in *S. alterniflora*.

In conclusion, this study establishes a valuable framework for the future investigation of key salt tolerance genes in a model halophyte. Moreover, it underscores the significance of ML-based annotation methods in accurately identifying gene functions on a whole-genome scale, particularly in under-studied species. Notably, the comparative transcriptomics between *S. alterniflora* and more extensively characterized model plant species unveiled a salt-responsive *SWEET* gene that is subject to convergent selection. These findings present a compelling set of candidate genes for potential use in genetic enhancement of salt tolerance in key fast-growing crops, thereby contributing to the advancement of sustainable food production practices.

## Materials and methods

### Observation of salt glands and determination of ion content

One-month-old seedlings underwent a 24-h exposure to diverse NaCl concentrations. Ion contents (Na^+^, K^+^, and Ca^2+^) were determined in 0.1 g of seedling roots or shoots using an inductively coupled plasma mass spectrometer (ICP-MS; SUPEC 7000 series, Hangzhou, China). Ion content determination involved 10 biological replicates. The ion content of salt crystals on the leaf surface was also analyzed by ICP-MS, employing 10 biological replicates of 0.1 g each.

### Transcriptomic sequencing

The RNA-seq data utilized in this study (Bioproject accession number PRJNA413596) were sourced from a previous report [22]. Transcriptomic data comprised raw datasets from 6-week-old *S. alterniflora* treated with 0, 350, 500, and 800 mM NaCl for 24 h, denoted as S0, S1, S2, and S3, respectively. Additionally, raw data were derived from wild-type rice (*O. sativa* L. ssp. *japonica* cv. ‘Nipponbare’) plants treated with 300 mM NaCl for 1, 5, or 24 h. Following quality control, trimming of adapter sequences, and removal of low-quality reads, high-quality RNA-seq reads from each library were aligned to our recently assembled *S. alterniflora* reference genome or the *O. sativa* ssp. *japonica* genome (IRGSP-1.0) using TopHat v2.0.14, following the parameters outlined by Kim *et al*. [57].

### Differentially expressed gene analysis, expression clustering, and qRT–PCR validation

Gene expression values were computed in fragments per kilobase of exon transcript per million mapped reads (FPKM). Reads uniquely mapped to reference sequences with no mismatches were employed in the analysis of DEGs. DESeq2 was utilized to classify genes as DEGs with thresholds of |log_2_(fold change)| ≥ 1 and a false discovery rate (FDR)-adjusted *P* < 0.01. Trend analysis, aimed at categorizing genes with similar change characteristic patterns within a changing trend, was conducted post-FPKM calculation. Expression patterns were clustered using the STEM [58] on the OmicShare platform (www.omicshare.com/tools). The analysis considered general types of gene expression patterns with a significance level of *P* < 0.05, and the number of trends was chosen to be nine.

Samples of plants treated with varying concentrations of NaCl (0, 350, 500, and 800 mM) were used for RNA isolation. Total RNA was extracted using the Trizol method. RNA quality was evaluated by agarose gel electrophoresis and quantity was determined using a Nanodrop spectrophotometer. Reverse transcription was performed using the Evo M-MLV kit to generate first-strand cDNA, which was then diluted 10-fold and used as a template for qRT–PCR. Specific primers were designed using NCBI Primer-BLAST and qRT–PCR was conducted using the SYBR Green kit (Accurate Biology, Changsha, China) following the manufacturer’s instructions. Each treatment had three biological replicates, and relative expression was calculated using the 2^−ΔΔCt^ method [59]. Results were analyzed and visualized in Excel 2010 and significant differences were analyzed using the LSD test at *P* < 0.05. Primers were synthesized by Sangon Biotech (Shanghai) and sequences are provided in Supplementary Data Table S12.

### Transcription factor prediction and Gene Ontology enrichment analyses

To identify TFs among the DEGs in *S. alterniflora*, each DEG was employed as a query in a BLAST search against the Plant Transcription Factor Database (PlantTFDB) using a threshold of *E*-value <10^−5^. DEGs were annotated with the protein language model NetGO 3.0 [7], following the method described in the source article, with all parameters set to the default threshold of the program. Annotations were categorized as enriched in specific gene sets at *P* < 0.05 (Fisher’s exact test) and an enrichment threshold of ≥1.5-fold higher than the background (the whole genome).

### Phylogenetic and convergent selection analyses

The protein sequences of various species were obtained from the Phytozome database. These sequences underwent alignment using T-Coffee [60]. Subsequently, the alignments were utilized to construct a phylogenetic tree in MEGA 7 [61] employing the neighbor-joining (NJ) method with 1000 bootstrap replicates. The protein sequence alignments and the phylogenetic tree served as input for Grand Convergence [62], a program that estimates the posterior number of convergent and divergent substitutions shared by all pairs of branches in the phylogenetic tree to identify convergent amino acid changes. Predicted convergent sites, classified as those with a probability >0.99, underwent manual examination to identify amino acid mutations shared by specific *S. alterniflora* genes and their orthologs. Utilizing the full-length transcripts from *S. alterniflora* and rice as input, a collinearity analysis was conducted in MCScanX with BLASTP v2.8.1, employing the following parameters: -evalue e-10 -num_alignments 5-outfmt 6.

### Alternative splicing detection and identification of salt stress-responsive alternative splicing events

rMATS v4.0.2 [63] was employed for the detection of differential alternative splicing (DAS) from replicate RNA-seq data. This program models exon inclusion levels while simultaneously evaluating the uncertainty of individual replicates and the variability between replicates, resulting in highly accurate validation of DAS events between conditions. The input for this analysis included RNA-seq BAM files and the GTF file containing the *S. alterniflora* reference genome. Various types of AS events (A3SS, A5SS, ES, RI, and MXE) were statistically validated with an FDR threshold of 0.05 to identify differences between isoform ratios.

## Acknowledgements

This work was financially supported by National Key R&D Program of China (2023ZD04073), the Nanfan special project, CAAS, Grant No. YBXM2304, and the Innovation Program of the Chinese Academy of Agricultural Sciences.

## Author contributions

H.H.L. conceived the original idea, designed the experiments, and provided overall supervision and direction of the work. Z.P.H., S.K.C., K.H.H., and T.X.Y. performed the experiments and data analysis. J.J.F. and S.G. provided valuable advice for the experimental design and optimization. Z.P.H., S.K.C., and H.H.L. wrote the manuscript. All authors discussed and commented on the manuscript.

## Data availability

All relevant data can be found within the paper and its supporting materials.

## Conflict of interest

The authors declare no conflicts of interest.

## Supplementary data

Supplementary data are available at *Horticulture Research* online.

## Supplementary Material

Web_Material_uhae082
