# Supplementary material for: Exploring salt tolerance mechanisms using machine learning for transcriptomic insights: case study in Spartina alterniflora
Source: Hortic Res. 2024 Mar 28;11(5):uhae082. doi: 10.1093/hr/uhae082 (PMC11101319; doi:10.1093/hr/uhae082)
Supplement: Web_Material_uhae082 [file web_material_uhae082.zip › Supplementary Figures.pdf]

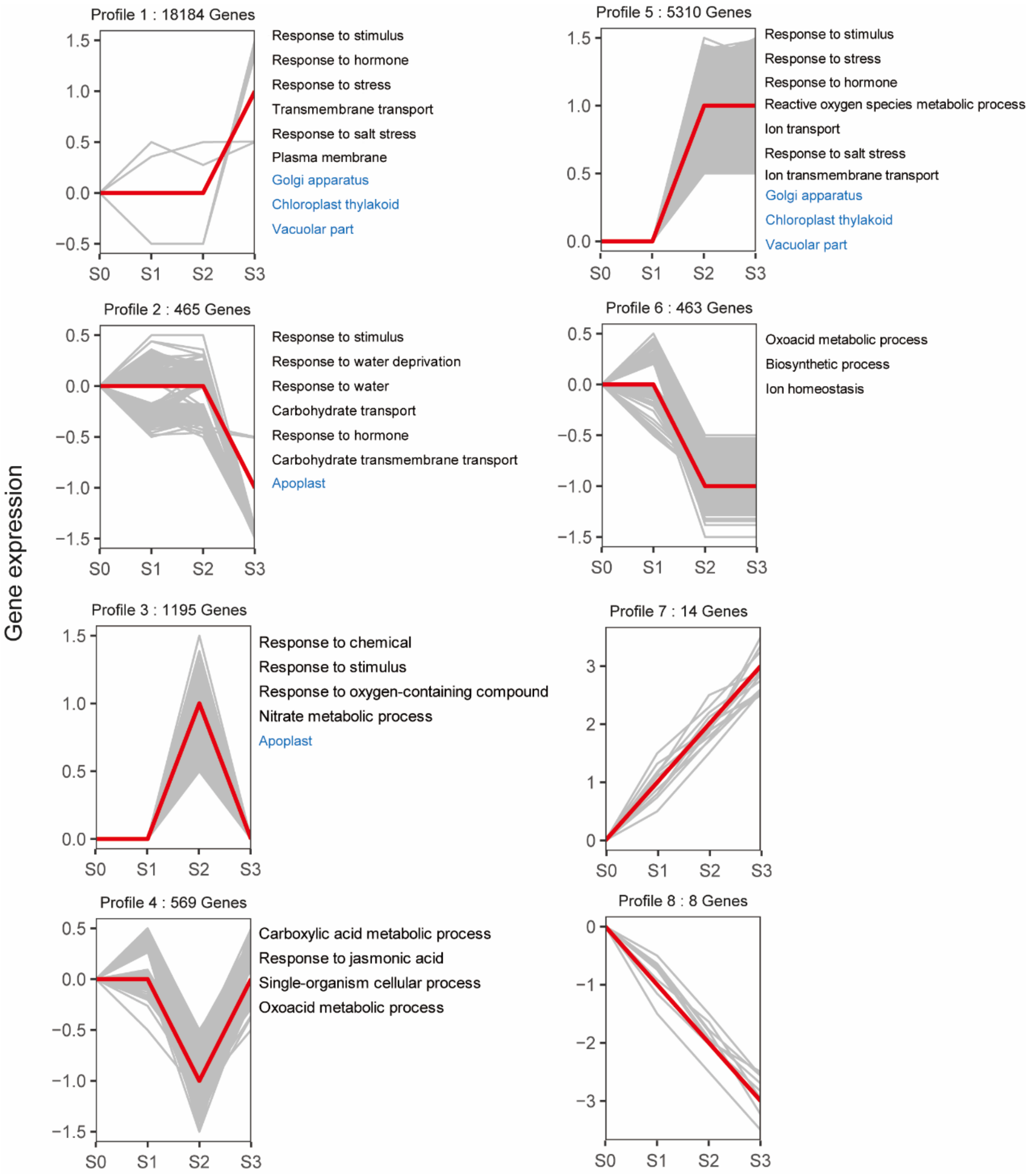

Supplementary Figure S1. Trend analysis of differentially expressed genes. The 28,721 DEGs in *S. alterniflora* in response to salt treatment were clustered into 9 profiles, and the profile displayed here were the top six of the total number of profiles. Each row in the figure represents the clustering results of each NaCl concentrations, and the legends in the right of the figures indicate the GO enrichments results of the corresponding profiles. The number on the upper left of the box denoted different trend clusters. Clusters 1, 2, 4, and 5 were the significant expression patterns (p-value < 0.05).

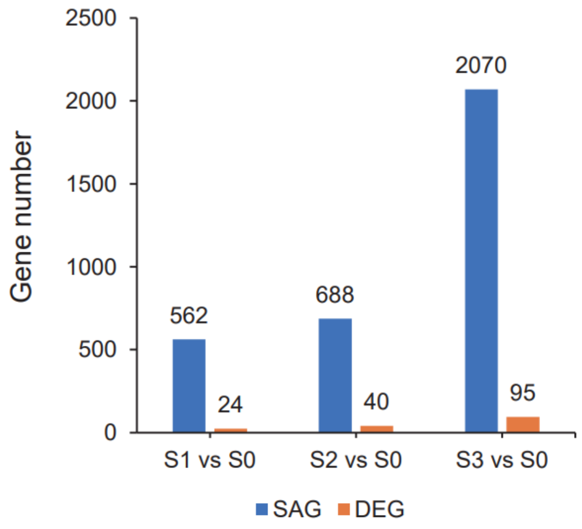

Supplementary Figure S2. Comparison analysis of differentially spliced (DAGs) and differentially expressed genes (DEGs) in response to salt stress.
